# The Complementary Roles of the Prostate Health Index and Multiparametric Magnetic Resonance Imaging in Optimizing the Detection of Clinically Significant Prostate Cancer in Patients With Prostate‐Specific Antigen Levels Below 10 Ng/mL

**DOI:** 10.1111/iju.70625

**Published:** 2026-09-14

**Authors:** Yoshitaka Sekine, Gaku Arai, Hiroki Kito, Mikio Sugimoto, Masaki Shiota, Hisashi Hasumi, Kazuto Ito, Yoshitomo Kobori, Takako Nakada, Takuma Kato, Akira Yokomizo, Yusuke Ito, Kazuhiro Suzuki, Yasushi Kaji

**Affiliations:** ^1^ Department of Urology, Graduate School of Medicine Gunma University Maebashi Japan; ^2^ Department of Urology Dokkyo Medical University Saitama Medical Center Koshigaya Japan; ^3^ JCHO Tokyo Shinjuku Medical Center Tokyo Japan; ^4^ Department of Urology Kagawa University Faculty of Medicine Kagawa Japan; ^5^ Department of Urology, Graduate School of Medical Sciences Kyushu University Fukuoka Japan; ^6^ Department of Urology and Renal Transplantation Yokohama City University Medical Center Yokohama Japan; ^7^ Institute for Preventive Medicine, Kurosawa Hospital Takasaki Japan; ^8^ Department of Radiology Shimane University, Faculty of Medicine Izumo Japan

**Keywords:** clinically significant prostate cancer, multiparametric magnetic resonance imaging, PI‐RADS, prostate biopsy, prostate health index

## Abstract

**Objective:**

To evaluate complementary roles of Prostate Health Index (phi) and multiparametric magnetic resonance imaging (mpMRI) in detection of any prostate cancer (PC) and clinically significant PC (csPC) and to investigate likelihood of risk stratification for recommending prostate biopsy in men with prostate‐specific antigen (PSA) ≤ 10 ng/mL.

**Methods:**

This exploratory post hoc analysis of the prospective multicenter PROPHET cohort included 108 Japanese men who underwent prebiopsy mpMRI. All MRI findings were retrospectively reviewed by one specialized radiologist and scored using Prostate Imaging Reporting and Data System (PI‐RADS) v2.1. csPC was defined as ISUP grade group (GG) of 2–5. Then, the likelihood of risk stratification for detecting any PC and csPC based on PI‐RADS categories and a significant [−2]proPSA‐related index assessed by receiver operating characteristic (ROC) analyses was retrospectively investigated.

**Results:**

PC was detected in 51 of 108 men (47.2%). The ROC analyses revealed the superiority of phi in detecting PC and csPC. Among men with phi ≤ 36.0 and PI‐RADS 1–3, csPC was detected in 2 of 36 men (5.6%), but no GG 3–5 cancers were observed. In contrast, in men with phi > 36.0, the risk of detecting PC/csPC was 42.9% (9/21)/23.8% (5/21), 42.9% (3/7)/42.9% (3/7), and 89.3% (25/28)/71.4% (20/28) in PI‐RADS 1–2, 3, and 4–5, respectively.

**Conclusions:**

phi and mpMRI have complementary roles for biopsy decision‐making in men with PSA ≤ 10 ng/mL. Men with phi ≤ 36.0 and PI‐RADS 1–3 may be candidates for biopsy avoidance, whereas phi > 36.0 may be a high‐risk group recommending prostate biopsy regardless of mpMRI findings.

**Registry and Registration No. of the Study/Trial:**

This study was registered with the **UMIN Clinical Trials Registry (UMIN‐CTR), registration number** UMIN000047187.

**Abbreviations**
[−2]proPSA: p2PSA
%p2PSA: p2PSA‐to‐free PSA ratio
AUC: area under the curve
csPC: clinically significant prostate cancer
GG: grade group
ISUP: International Society of Urological Pathology
mpMRI: multiparametric magnetic resonance imaging
MRI: magnetic resonance imaging
MRI‐TB: magnetic resonance imaging‐targeted biopsy
PC: prostate cancer
phi: Prostate Health Index
PI‐RADS: Prostate Imaging Reporting and Data System
PPV: positive predictive value
PSA: prostate‐specific antigen
ROC: receiver operating characteristic
ROI: region of interest
SB: systematic biopsy
TRUS: transrectal ultrasound

## 1 Introduction

Prostate‐specific antigen (PSA) is widely used for the early detection of prostate cancer (PC). However, particularly in patients with PSA levels of 4–10 ng/mL, prostate biopsy has low specificity and a relatively low cancer detection rate, which can lead to unnecessary biopsies [1]. According to multicenter studies and systematic reviews, Prostate Health Index (phi), a [−2]proPSA (p2PSA)‐based biomarker, has better discriminating power in the detection of clinically significant PC (csPC) than PSA alone or the %f‐PSA (free‐to‐total PSA ratio), while maintaining higher specificity [2, 3, 4, 5].

On the other hand, prostate magnetic resonance imaging (MRI) has seen advances in standardizing imaging diagnosis through the Prostate Imaging Reporting and Data System (PI‐RADS), improving the detection accuracy of csPC. Regarding the latest version, PI‐RADS v2.1, systematic reviews and meta‐analyses have reported its superior cancer detection rate [6, 7]. However, from the perspective of determining prostate biopsy indications, the optimal combination of serum markers and MRI (e.g., which PI‐RADS categories or phi thresholds should recommend or avoid biopsy), particularly in MRI‐negative cases or those with borderline phi values, remains unclear. Indeed, the combined use of phi and MRI has been demonstrated to be superior to either modality alone in detecting csPC and to improve triage performance in cases where biopsy is uncertain, such as PI‐RADS 3. However, specific cutoff values and operational details of complementary use (in the order using phi or MRI, or criteria for integrated use) vary across studies [8, 9, 10].

We have previously demonstrated, using real‐world clinical data from Japan, the complementary relationship between phi and PI‐RADS and the potential for avoiding biopsies [11]. However, the verification regarding the risk of missing csPC in MRI‐negative cases (PI‐RADS 1–2) and the complementary role of phi, as well as the complementary role of MRI in low‐to‐moderate phi regions, remains insufficient. Therefore, a post hoc PROPHET‐MRI study was conducted to evaluate the likelihood of missing csPC in MRI‐negative cases and the complementary role of phi retrospectively, as well as the likelihood of missing csPC below the phi cutoff value and the complementary role of MRI findings, using men undergone multiparametric MRI (mpMRI) in the PROPHET cohort [4]. Then, we preliminarily propose a specific combined strategy to aid in recommending prostate biopsy in the patient group with PSA ≤ 10 ng/mL.

## 2 Methods

### 2.1 Study Design and Participants

The PROPHET‐MRI is a retrospective subanalysis using data from the PROPHET study, a prospective multicenter cohort study conducted in Japan [4]. Of the 421 men enrolled in PROPHET between April 2015 and March 2017, 108 underwent mpMRI before prostate biopsy. All participants met the original PROPHET eligibility criteria, briefly, the age was between 50 and 79 years, serum PSA at each institution was between the age‐stratified PSA cutoffs (i.e., 3.0 ng/mL in the age 50 to 64 years, 3.5 ng/mL in 65 to 69 years, 4.0 ng/mL in 70 years or older) and 10 ng/mL, and underwent an initial prostate biopsy within three months of enrollment.

### 2.2 MRI Acquisition and Central Imaging Review

All MRI examinations were performed before prostate biopsy at each participating institution. For this study, one experienced radiologist (Y.K.), with more than 30 years of experience in diagnostic radiology, centrally reviewed the digital imaging data while blind to the clinical and pathological findings. PI‐RADS v2.1 scores were assigned based on standardized mpMRI sequences, including T2‐weighted imaging, diffusion‐weighted imaging, and dynamic contrast‐enhanced imaging. Using central imaging assessment ensured uniformity of PI‐RADS interpretation across institutions.

### 2.3 Prostate Biopsy and Pathological Evaluation

All participants underwent a systematic ultrasound‐guided biopsy (SB) consisting of 12–20 cores. According to the PROPHET protocol, targeted biopsies could additionally be performed for hypoechoic lesions detected on transrectal ultrasound (TRUS) and for MRI‐suspicious lesions identified by local radiologists using cognitive MRI‐targeted biopsy (MRI‐TB); however, no standardized common template was used for systematic biopsy. In the present study, we retrospectively judged that the region of interest (ROI) of MRI evaluated by a central radiologist had been biopsied if biopsy sites were located within 1 cm of the center of the ROI. Pathological results were reported according to the 2014 ISUP Grade Group system (ISUP GG) by pathologists at each institution.

### 2.4 Laboratory Measurements

Serum total PSA, free PSA, and p2PSA were measured using the Access Hybritech assay on the UniCel DxI 800 platform, consistent with the original PROPHET protocol. phi and %p2PSA (p2PSA‐to‐free PSA ratio) were calculated using standard formulas: phi = (p2PSA/free PSA) × √(total PSA), and %p2PSA = (p2PSA/free PSA) × 100.

### 2.5 Statistical Analysis

Continuous variables (age, PSA, phi, and %p2PSA) were compared between groups using the Mann–Whitney U test. The diagnostic performance for detecting PC was assessed using receiver operating characteristic (ROC) curves. The area under the ROC curve (AUC) was calculated for PSA, phi, and %p2PSA. Statistical significance was set at *p* ≤ 0.05. All analyses were performed using SPSS Statistics, version 29 (IBM Corp., IL, USA).

### 2.6 Institutional Ethics Approval

This study was approved by the Ethics Committee of Gunma University (Approval No. 1962) and the institutional review boards of all participating centers (UMIN000047187). Written informed consent for participation was not required for this study, as it was conducted under an opt‐out consent procedure.

## 3 Results

### 3.1 Patient Characteristics

**FIGURE 1:**
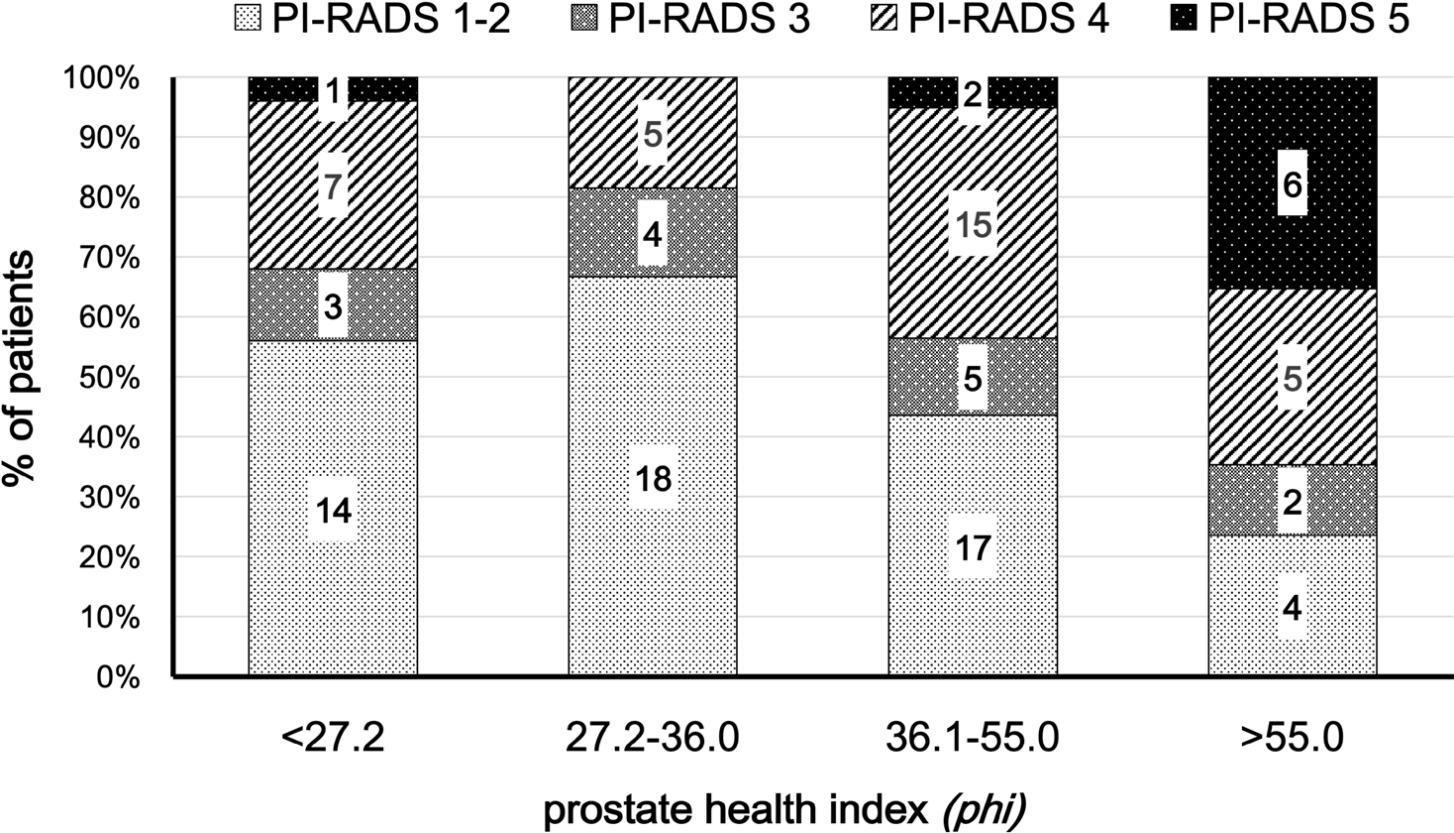
Distribution of Prostate Imaging Reporting and Data System (PI‐RADS) categories (1–2, 3, and 4–5) across prostate health index (phi) categories (< 27.2, 27.2–36.0, 36.1–55.0, and > 55.0).

Tables S1 and S2 provide a comprehensive overview of the clinical characteristics of the 108 patients who were included in this study. A comprehensive analysis of the central laboratory data revealed a median PSA of 5.91 ng/mL, a median %p2PSA of 1.51, and a median phi of 37.10. The distribution of phi values was as follows: < 27.2 in 25 cases (23%), between 27.2 and 36.0 in 27 cases (25%), between 36.1 and 55.0 in 39 cases (36%), and > 55.0 in 17 cases (16%). The PI‐RADS scores were distributed as follows: 1–2 in 53 cases (49%), 3 in 14 cases (13%), 4 in 32 cases (30%), and 5 in 9 cases (8%). Of the 108 cases examined, 51 (47.2%) were found to have PC. In comparison with the non‐PC group, the PC group was found to be older and exhibited significantly elevated PSA, %p2PSA, and phi levels (all *p* < 0.001). Furthermore, patients with csPC, defined as GG 2–5, were significantly older and had significantly higher PSA, %p2PSA, and phi levels than those in the non‐PC + GG1 group (all *p* < 0.05; Table S2). The distribution of PI‐RADS categories within each phi is illustrated in Figure 1. Patients with higher phi exhibited a higher proportion of PI‐RADS 4–5 findings, which was 64.7% in phi > 55.0.

### 3.2 Prediction of Prostate Cancer

Table 1 summarizes the diagnostic performance of PSA, phi, and %p2PSA for any PC, ISUP GG 2–5, and ISUP GG 3–5 detection. In concern with distinguishing csPC from non‐csPC, the AUC‐ROC and specificity, maintaining 90% sensitivity, respectively, were 0.670 and 45.8% for PSA, 0.774 and 30.6% for phi, and 0.706 and 29.2% for %p2PSA. phi demonstrated the highest AUC‐ROC in all the above discriminations.

**TABLE 1:** AUC‐ROC and specificity at 95%, 90%, 80%, and 70% sensitivity.

Specificity at			Specificity at	Specificity at	Specificity at
Index	AUC‐ROC (95% CI)	95% sensitivity	90% sensitivity	80% sensitivity	70% sensitivity
Non‐PC vs. PC					
PSA	0.691 (0.591–0.792)	15.8%	29.8%	49.1%	61.4%
*phi*	0.758 (0.668–0.849)	24.6%	31.6%	52.6%	70.2%
%p2PSA	0.695 (0.597–0.794)	15.8%	33.3%	43.9%	56.1%
Non‐PC + GG1 PC (non‐csPC)					
vs. GG2‐5 PC (csPC)					
PSA	0.670 (0.565–0.774)	12.5%	45.8%	52.8%	59.7%
*phi*	0.774 (0.677–0.870)	23.6%	30.6%	59.7%	76.4%
%p2PSA	0.706 (0.601–0.811)	25.0%	29.2%	47.2%	52.8%
Non‐PC + GG1‐2 PC					
vs. GG3‐5 PC					
PSA	0.662 (0.546–0.778)	11.1%	11.1%	40.7%	65.4%
*phi*	0.804 (0.708–0.900)	25.9%	37.0%	74.1%	80.2%
%p2PSA	0.734 (0.626–0.842)	25.9%	28.4%	51.9%	63.0%

Abbreviations: AUC, area under the curve; CI, confidence interval; GG, ISUP grade group; PC, prostate cancer; phi, prostate health index; PSA, prostate‐specific antigen; ROC, receiver operating characteristic.

### 3.3 Stratification Utilizing Phi and PI‐RADS

**FIGURE 2:**
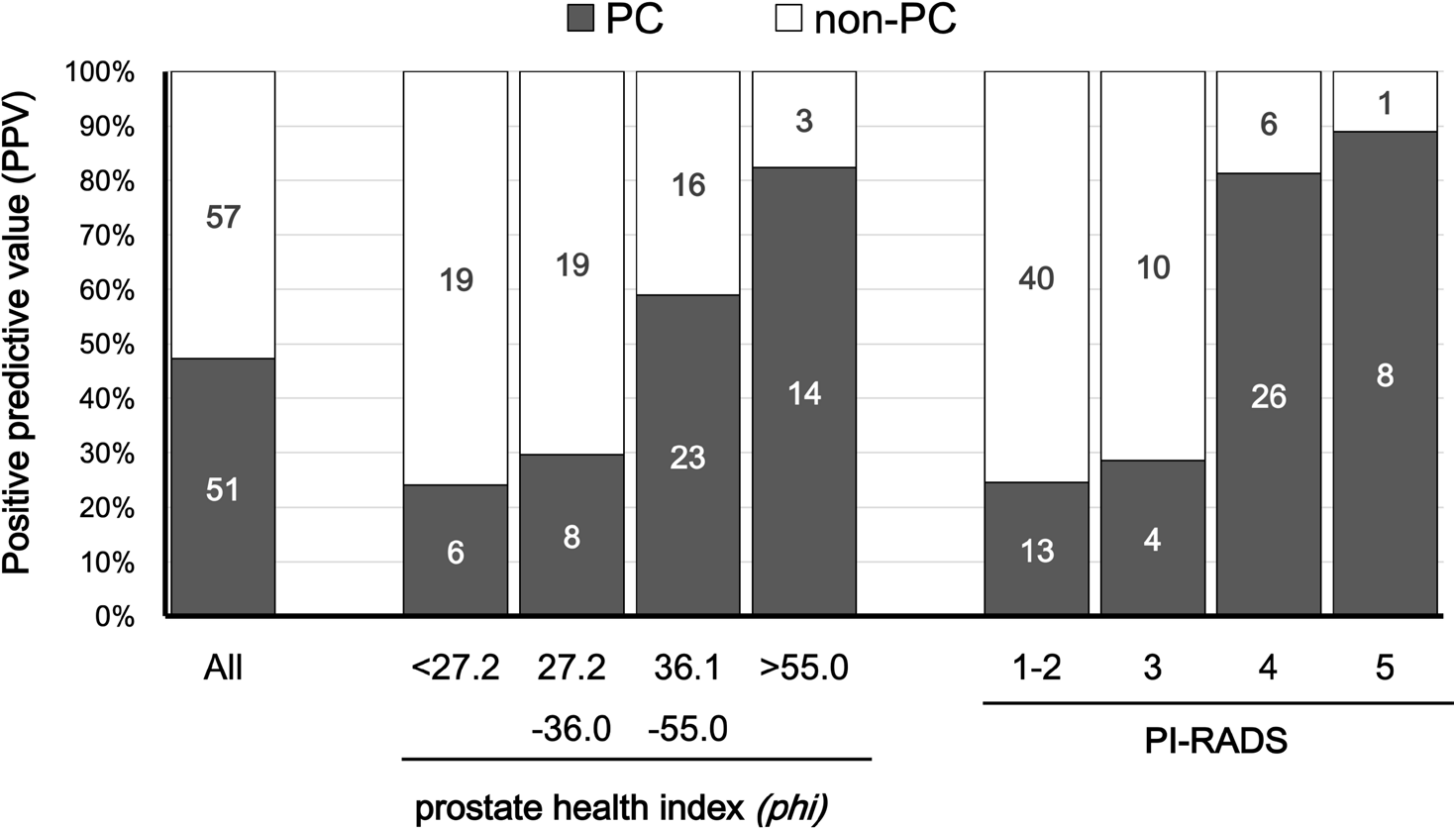
Positive predictive value (PPV) for detection of any prostate cancer stratified by prostate health index (phi) categories (< 27.2, 27.2–36.0, 36.1–55.0, and > 55.0) and Prostate Imaging Reporting and Data System (PI‐RADS) categories.

**FIGURE 3:**
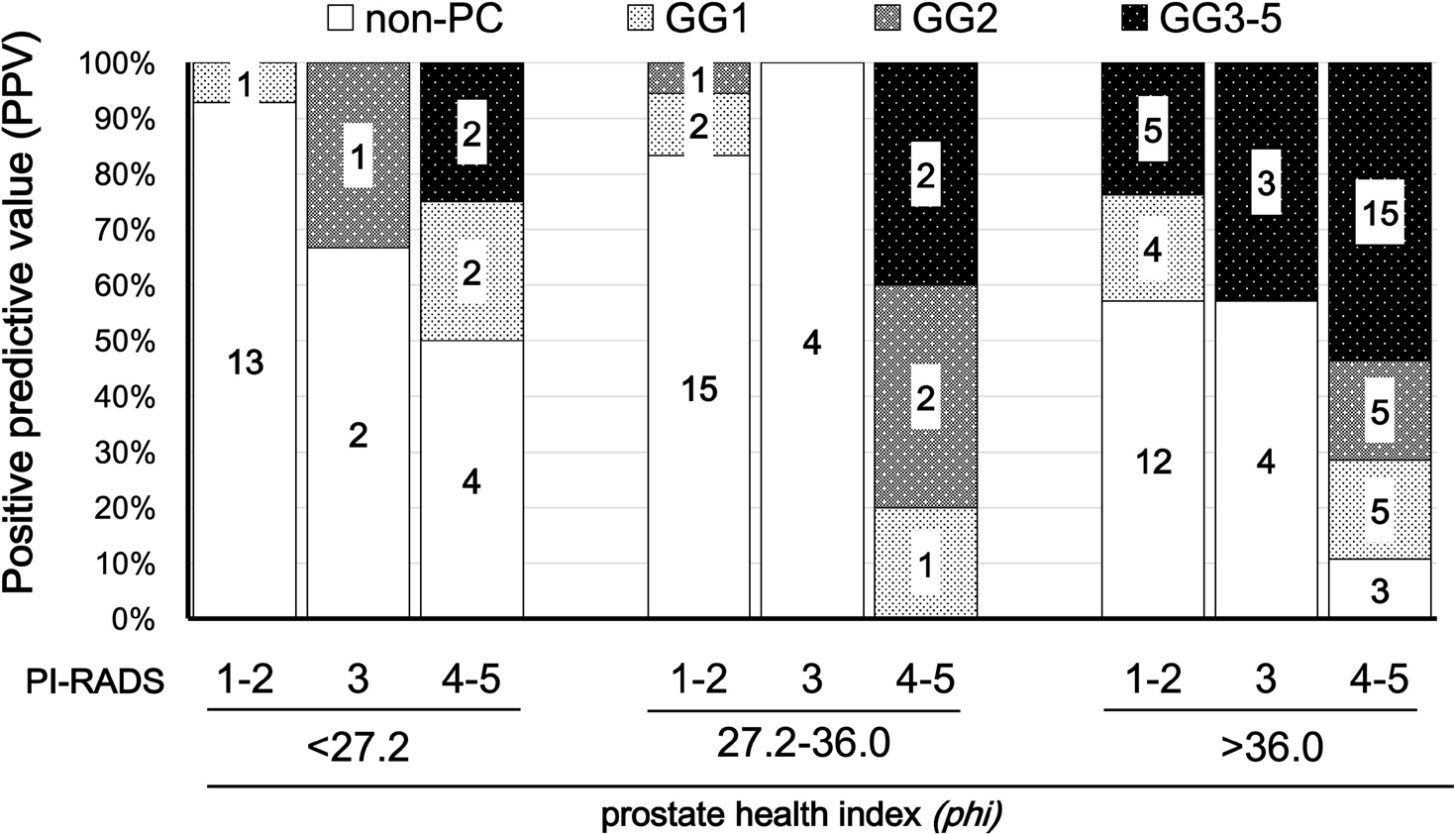
Risk stratification for prostate cancer and clinically significant prostate cancer using prostate health index and PI‐RADS. PC, prostate cancer; GG, International Society of Urological Pathology grade groups; PI‐RADS, Prostate Imaging Reporting and Data System.

Figure 2 shows the positive predictive value (PPV) of PC by phi and PI‐RADS categories. PPV increased stepwise with rising phi. Similarly, PPV increased with rising PI‐RADS categories. Among 51 patients diagnosed with PC, the ISUP GG distribution was GG1 in 29%, GG2 in 18%, GG3 in 24%, GG4 in 20%, and GG5 in 10% (Figure S1). GG5 was only observed in men in the high‐risk group with phi > 36.0 and PI‐RADS 4–5. When combining phi and PI‐RADS, the likelihood of having any PC or high‐grade PC was able to be stratified more clearly (Figure 3). In the phi < 27.2 group, the PPV of PC was particularly low at 7.1% (1/14) in patients with PI‐RADS 1–2, and the single detected cancer was ISUP GG1. Furthermore, even within the phi ≤ 36.0 group, the PPV of PC was still low at 12.5% (4/32) in patients with PI‐RADS 1–2 and 14.3% (1/7) in those with PI‐RADS 3, whereas it increased to 69.2% (9/13) in those with PI‐RADS 4–5. In the phi > 36.0 group, the PPV of PC increased further, which reached 89.3% (25/28) for PI‐RADS 4–5.

When considering the probability of csPC in the phi ≤ 36.0 groups, only two cases (5.1% in 39 cases) were observed in men with PI‐RADS 1–3, while it increased to 46.2% (6/13) in PI‐RADS 4–5. In the phi > 36.0 group, the probability of csPC accounted for 28.6% (8/28) in PI‐RADS 1–3 and 71.4% (20/28) in PI‐RADS 4–5. When concerning detection of ISUP GG3‐5 in the phi ≤ 36.0, no GG3‐5 disease was observed with PI‐RADS ≤ 3, whereas it accounted for 30.8% (4/13) in patients with PI‐RADS 4–5. In patients with phi > 36.0, PPV was high at 28.6% (8/28) even in PIRADS 1–3 and increased to 53.6% (15/28) in PI‐RADS 4–5. Because several subgroups contained very few patients (e.g., 7–13 cases), these proportions are statistically unstable and should be interpreted as descriptive and exploratory only.

## 4 Discussion

phi is a marker calculated from total PSA, free PSA, and p2PSA. Numerous Western cohorts have demonstrated its ability to detect csPC with higher specificity than PSA [3, 12, 13]. The AUC‐ROC values for phi in the PROPHET‐MRI (0.758) are consistent with the total cohort of the PROPHET study and prior reports [14, 15], reaffirming that phi consistently demonstrates high diagnostic performance even in Japanese men who have a lower incidence of PC. Furthermore, in this analysis, the probability of PC and csPC increased stepwise with rising phi. This indicates that phi is useful not only for deciding “to biopsy or not,” but also for risk stratification, specifically, “how strongly csPC should be suspected,” consistent with previous reports [16, 17].

While mpMRI contributes to improved csPC detection rates and reduced unnecessary biopsies, challenges remain, including the management of PI‐RADS 3 lesions and MRI‐negative cases, as well as inter‐facility and inter‐observer variability [18]. According to the meta‐analysis including 15 to 20 first biopsy series, detection rates of csPC were 44.2% and 8.1%, respectively, in MRI‐positive and MRI‐negative cohorts [19]. Regarding the combination of phi and mpMRI, several studies investigated the role of phi on biopsy indications in men with PI‐RADS 1–2 [10, 11, 20, 21]. A large retrospective study held in Hong Kong demonstrated that the probability of csPC was low in men with PI‐RADS 1–2 and low phi values, which was 0% (0/26) and 5.0% (1/20) in phi < 25.0 and 25.0–34.9, respectively [21].

The PROPHET‐MRI retrospectively evaluated the complementary roles of phi and mpMRI as a post hoc analysis of the multicenter prospective PROPHET study. As indicated in Figure 3, combining phi and MRI enabled detection of ISUP GG3‐5 PC cases that must not be missed among men with PI‐RADS 1–3. Even in men with PI‐RADS 1–3, PPV for detecting csPC was high at 28.6% (8/28) in patients with phi > 36.0; thus, SB should be recommended. On the other hand, in cases with low phi ≤ 36.0, prostate biopsy may be able to be avoided in men with low PI‐RADS scores between 1 and 3 because the likelihood of detecting ISUP GG2 and GG3‐5 PC, respectively, was extremely low at 5.1% (2/39) and 0% (0/39), but MRI‐TB and SB would be recommended in men with PI‐RADS 4–5 because the likelihood of detecting ISUP GG2‐5 and GG3‐5 PC respectively increased to 46.2% (6/13) and 30.8% (4/13).

The strengths of this study include the following points. First, participants in this post hoc analysis were selected from the database participated in the multicenter, prospective PROPHET cohort of Japanese men with PSA < 10 ng/mL who underwent MRI before phi being apparent. Therefore, it may be possible to evaluate complementary roles of phi and mpMRI in a population close to real‐world clinical practice. Second, laboratory samples were ideally stored at −70°C immediately after serum separation in all institutions participating in the PROPHET, and p2PSA and phi were measured at the central laboratory at Beckman Coulter, thereby minimizing inter‐institutional measurement variability. Third, mpMRI images were centrally read by a single specialized radiologist using PI‐RADS v2.1, reducing the impact of inter‐reader variability. This allowed risk stratification using the combination of serum biomarkers and imaging to be evaluated under relatively homogeneous conditions. Therefore, this preliminary study might have been able to propose a specific biopsy strategy for detecting not only PC but also csPC by combining the phi cutoff of 36.0 with the PI‐RADS score 1–3 and 4–5.

On the other hand, this retrospective study also has several limitations. First, the study population was limited to 108 cases from the PROPHET registry who underwent MRI, and clinical selection bias may have influenced MRI implementation. However, 76% (82/108) cases were done at the top two institutions among 7 participating institutions in the PROPHET. Furthermore, the percentage of men undergoing MRI in the top two institutions was very high at 96% (82/85). Therefore, the likelihood of selection bias by each investigator may be modest. Second, biopsies were primarily performed using systematic TRUS‐guided biopsy, with cognitive MRI‐TB added in selected cases. Because targeted biopsy was not mandatory under the PROPHET protocol, the biopsy strategy may differ from contemporary MRI–ultrasound fusion‐targeted biopsy approaches, and the cohort included cases in which MRI‐defined ROI were not sampled. Nevertheless, a retrospective review of 38 PC cases with PI‐RADS 3–5 lesions showed that biopsy cores had been obtained from the ROI identified by a central radiologist in 32 cases (84%). Third, the phi cutoff of 36.0 was derived partly through exploratory analysis of the same dataset, and the reported performance is therefore susceptible to optimistic bias. Fourth, pathological diagnoses were made by pathologists at each participating institution without central pathological review. Fifth, because of the limited number of events, this study was descriptive and exploratory and was not designed to establish whether phi provides predictive information independent of or incremental to PI‐RADS. The proposed combined strategy should be regarded as hypothesis‐generating and requires confirmation in larger prospective cohorts.

In conclusion, the PROPHET‐MRI in Japanese men with PSA < 10 ng/mL demonstrated that phi combined with mpMRI enables more precise risk stratification for recommending prostate biopsy. Specifically, cases with phi ≤ 36.0 and PI‐RADS 1–3 showed a low missing rate of csPC at 5.6%, making them potential candidates for deferring immediate biopsy, although these exploratory findings require prospective external validation before clinical application. Conversely, cases with phi > 36.0 should be considered high‐risk groups warranting active biopsy consideration, regardless of MRI findings. Future studies should prospectively evaluate the biopsy strategy proposed here in larger, more heterogeneous cohorts to develop and validate a phi‐ and mpMRI‐based PC diagnostic algorithm.

## Author Contributions

**Yoshitaka Sekine:** conceptualization, methodology, formal analysis, data curation, visualization, writing – original draft, writing – review and editing. **Kazuto Ito:** conceptualization, methodology, formal analysis, supervision, writing – review and editing. **Masaki Shiota:** data curation, writing – review and editing. **Gaku Arai:** data curation. **Yasushi Kaji:** data curation, investigation, formal analysis. **Yusuke Ito:** data curation. **Takako Nakada:** data curation. **Hiroki Kito:** data curation. **Hisashi Hasumi:** data curation. **Mikio Sugimoto:** data curation. **Takuma Kato:** data curation. **Yoshitomo Kobori:** data curation. **Akira Yokomizo:** data curation. **Kazuhiro Suzuki:** supervision, conceptualization.

## Funding

This work was supported by the present study was conducted with financial support from Beckman Coulter Inc. to the Clinical Research Support Center Kyushu (Fukuoka, Japan).

## Ethics Statement

This study was approved by the Ethics Committee of Gunma University (Approval No. 1962).

## Consent

An opt‐out approach was used to obtain informed consent from patients.

## Conflicts of Interest

Mikio Sugimoto and Kazuhiro Suzuki are Editorial Board members of the International Journal of Urology and co‐authors of this article. To minimize bias, they were excluded from all editorial decision‐making related to the acceptance of this article for publication. No competing interests are declared in relation to this study.

## Supporting information


**Figure S1:** Distribution of International Society of Urological Pathology grade groups (GG 1–5) among patients diagnosed with prostate cancer. PI‐RADS, Prostate Imaging Reporting and Data System.

**Table S1:** Participant clinical background.

**Table S2:** Clinicopathological findings in participants with vs. without prostate cancer.

## Data Availability

The data that support the findings of this study are available from the corresponding author upon reasonable request.
