# Supplementary material for: The Complementary Roles of the Prostate Health Index and Multiparametric Magnetic Resonance Imaging in Optimizing the Detection of Clinically Significant Prostate Cancer in Patients With Prostate‐Specific Antigen Levels Below 10 Ng/mL
Source: Int J Urol. 2026 Sep 14;33(9):e70625. doi: 10.1111/iju.70625 (PMC13574299; doi:10.1111/iju.70625)
Supplement: Supplementary file 1 [file IJU-33-0-s001.pdf]

Supplemental table 2. Clinicopathological findings in participants with vs without prostate cancer

	PC	non-PC	p value
No. pts	51	57	
Mean ± SD age	69.20 ± 5.82	64.43 ± 6.80	<0.001*
Mean ± SD PSA (ng/ml)	7.12 ± 2.00	5.82 ± 2.19	<0.001*
Mean ± SD %p2PSA	1.99 ± 1.43	1.35 ± 0.46	<0.001*
Mean ± SD *phi*	53.40 ± 44.31	31.41 ± 10.91	<0.001*
phi (%)			
<27.2	6 (12)	19 (33)	
27.2-36.0	8 (16)	19 (33)	
36.1-55.0	23 (45)	16 (28)	
>55.0	14 (27)	3 (5)	
PI-RADS (%)			
1-2	13 (25)	40 (70)	
3	4 (8)	10 (18)	
4	26 (51)	6 (11)	
5	8 (16)	1 (2)	
No. Grade Group (%)			
1	15 (29)		
2	9 (18)		
3	12 (24)		
4	10 (20)		
5	5 (10)		

PC; prostate cancer, SD; standard deviation, PSA; prostate specific antigen, *phi*; prostate health index, PI-RADS; Prostate Imaging Reporting and Data System

Clinical laboratory-based data were measured at central laboratory.

* Mann-Whitney U test.

Percentages may not total 100% due to rounding.
