# Supplementary material for: The Complementary Roles of the Prostate Health Index and Multiparametric Magnetic Resonance Imaging in Optimizing the Detection of Clinically Significant Prostate Cancer in Patients With Prostate‐Specific Antigen Levels Below 10 Ng/mL
Source: Int J Urol. 2026 Sep 14;33(9):e70625. doi: 10.1111/iju.70625 (PMC13574299; doi:10.1111/iju.70625)
Supplement: Supplementary file 2 [file IJU-33-0-s002.pdf]

Supplemental table 1. Participant clinical background

Mean ± SD age (range) / median (IQR)	66.7 ± 6.76 (50–78) / 67.5 (62.9-72.0)
Mean ± SD PSA at each institution, ng/ml (range) / median (IQR)	6.06 ± 1.74 (3.40–10.00) / 5.77 (4.69–7.09)
Central laboratory data (Mean ± SD (range) / median (IQR)	
PSA (ng/ml)	6.44 ± 2.16 (2.44–11.90) / 5.91 (4.79–7.88)
free PSA (ng/ml)	1.05 ± 0.50 (0.30–2.68) / 0.96 (0.67–1.32)
p2PSA (pg/ml)	16.26 ± 11.32 (3.92–83.62) / 13.43 (8.98–18.91)
%p2PSA	1.65 ± 1.08 (0.38-10.21) / 1.51 (1.16–1.84)
phi	41.79 ± 33.20 (9.40–315.30) / 37.10 (27.30–43.80)
phi (%)	
<27.2	25 (23%)
27.2-36.0	27 (25%)
36.1-55.0	39 (36%)
>55.0	17 (16%)
PI–RADS (%)	
1-2	53 (49%)
3	14 (13%)
4	32 (30%)
5	9 (8%)

SD; standard deviation, IQR; interquartile range, PSA; prostate specific antigen, *phi*; prostate health index, PI-RADS; Prostate Imaging Reporting and Data System
